# Hyperspectral estimation of magnesium content in Yunyan 87 and Zhongyan 100 tobacco leaves using machine learning

**DOI:** 10.3389/fpls.2026.1777447

**Published:** 2026-03-26

**Authors:** Beibei Li, Qian Zhang, Zekai Wang, Yike Li, Hanjun Zhou, Xiefeng Ye

**Affiliations:** 1 Key Laboratory of Tobacco Cultivation of Tobacco Industry, National Tobacco Cultivation and Physiology and Biochemistry Research Centre, Tobacco Science College of Henan Agricultural University, Zhengzhou, China; 2 Bozhou Branch Company of Zunyi Tobacco Company, Zunyi, China

**Keywords:** flue-cured tobacco, hyperspectral remote sensing, machine learning models, magnesium content, non-destructive estimation

## Abstract

Hyperspectral remote sensing provides a rapid and non-destructive approach for monitoring plant nutrient status; however, its application for magnesium (Mg) estimation in flue-cured tobacco remains limited. In this study, two cultivars, Yunyan 87 and Zhongyan 100, were grown in a hydroponic system with five Mg concentration gradients (0, 0.2, 1, 5, and 25 mmol L^-1^). Hyperspectral reflectance data of fresh leaves were collected at different growth stages. Three preprocessing methods, including first derivative (FD), standard normal variate (SNV), and multiplicative scatter correction (MSC), were applied, and partial least squares regression (PLSR) was used to identify the optimal preprocessing strategy. Characteristic wavelengths were selected using competitive adaptive reweighted sampling (CARS), successive projections algorithm (SPA), and genetic algorithm (GA), and were further combined with extreme learning machine (ELM), support vector regression (SVR), and radial basis function (RBF) neural network models to estimate Mg content. The results showed that spectral preprocessing significantly improved the relationship between hyperspectral data and Mg content, with optimal methods varying across cultivars and growth stages. Selected wavelengths were mainly located in the near-infrared region. The developed models achieved high prediction accuracy, particularly during the middle and late growth stages, where the coefficients of determination (R^2^) of all test sets exceeded 0.90. In addition, Yunyan 87 exhibited higher prediction accuracy than Zhongyan 100. These findings demonstrate that hyperspectral technology combined with feature wavelength selection and machine learning enables accurate and non-destructive estimation of Mg content in flue-cured tobacco leaves, providing a reliable tool for Mg nutrition diagnosis and precision management. However, further validation under diverse field conditions is required to enhance model robustness.

## 1 Introduction

Magnesium (Mg), as an activator of numerous enzymes and a core constituent of chlorophyll molecules, plays a critical role in photosynthesis, carbon–nitrogen metabolism, and the synthesis of secondary metabolites in flue-cured tobacco (Cakmak and Kirkby, 2008). Both Mg deficiency and excess can induce physiological and metabolic disorders in flue-cured tobacco, thereby adversely affecting leaf appearance, internal chemical composition, and sensory smoking quality (Zhang et al., 2009; Zhu et al., 2011). In southern China, high temperatures, abundant rainfall, and acidic soils promote Mg migration and leaching losses (Yuan et al., 2011). Moreover, with the prolongation of tobacco cultivation, soil-available Mg content has shown a continuous decline, and Mg deficiency symptoms have been observed in some tobacco-growing regions (Ran, 1986). Therefore, monitoring leaf Mg content is essential for evaluating Mg nutritional status and guiding rational Mg fertilization (Jia et al., 2018).Traditionally, the monitoring of Mg nutritional status in flue-cured tobacco mainly relies on soil and plant chemical analyses (He et al., 2012). Soil chemical analysis can reflects the potential Mg supply capacity but cannot directly characterize Mg uptake and utilization by plants, while plant chemical analysis, although providing direct information on Mg nutritional status, is time-consuming, costly, and destructive, making it unsuitable for large-scale and rapid monitoring (Li, 2013). Therefore, there is an urgent need to develop rapid, accurate, and efficient monitoring methods to meet the requirements of precision nutrient management in tobacco production. Hyperspectral remote sensing technology, as an advanced non-destructive approach, is characterized by high spectral resolution, spectral continuity, and abundant information content, providing the possibility for rapid and non-destructive acquisition of vegetation physiological parameters and offering a new technical pathway for precision nutrient regulation in tobacco fields.

Hyperspectral remote sensing technology, as an advanced non-destructive approach, is characterized by high spectral resolution, spectral continuity, and abundant information content, providing the possibility for rapid and non-destructively acquisition of vegetation physiological parameters and offering a new technical pathway for precision nutrient regulation in tobacco fields (Cheng and Hu, 2001; Zhang and Zheng, 2013). Numerous studies have demonstrated that hyperspectral data can effectively estimate key physiological indicators such as nitrogen, chlorophyll, and starch in crops, providing important technical support for precision agricultural management (Jia et al., 2013; Cao et al., 2021; Guo et al., 2021; Zhang et al., 2024; Hou et al., 2025; Zhang et al., 2025). However, compared with studies on nitrogen and other nutrients, hyperspectral inversion research on Mg content in flue-cured tobacco remains limited.

Field conditions introduce variability in soil properties, climate, and management practices, under which leaf Mg content in flue-cured tobacco is influenced by multiple interacting environmental factors, complicating precise control of Mg stress gradients and hindering accurate characterization of the relationship between spectral features and Mg content. This complexity further limits the stability and broader applicability of Mg content estimation models under field conditions. To address these challenges, this study employed a hydroponic experimental system to systematically generate tobacco samples under controlled Mg nutritional levels, thereby effectively eliminating soil-related environmental interference and enabling precise regulation of Mg nutritional status. This approach allows a clear investigation of the intrinsic spectral response mechanisms between Mg content and hyperspectral characteristics and facilitates the development of high-precision inversion models, providing a reliable theoretical basis and model foundation for subsequent field applications. The study focused on two flue-cured tobacco cultivars, Yunyan 87 and Zhongyan 100, as representative genotypes for Mg nutrition assessment. Hyperspectral techniques were used to estimate leaf Mg content, analyze correlations between Mg content and hyperspectral features, and construct predictive models, aiming to establish a rapid and non-destructive estimation framework for Mg nutrition diagnosis. The results are expected to provide a reference framework for rapid and non-destructive estimation of Mg content in flue-cured tobacco, and to lay a foundation for future validation and application under field conditions.

## 2 Materials and methods

### 2.1 Experimental methods

The experiment was conducted from March to July 2024 at the National Research Base for Tobacco Cultivation, Physiology, and Biochemistry, Henan Agricultural University (34°08′ N, 113°48′ E). The flue-cured tobacco cultivars Yunyan 87 and Zhongyan 100 were used as experimental materials. According to previous studies (Pang and Wu, 2015), tobacco plants exhibit normal growth without visible deficiency or toxicity symptoms when the magnesium concentration in the nutrient solution ranges from 0.4 to 8 mmol·L^-^¹, whereas Mg concentrations ≥ 20 mmol·L^-^¹ induce toxicity symptoms. Based on these findings, five Mg concentration treatments were established: T0, T1, T2, T3, and T4, corresponding to 0, 0.2, 1, 5, and 25 mmol·L^-^¹, respectively. These treatments represented three Mg nutritional levels: low Mg (0, 0.2 mmol·L^-^¹), normal Mg (1, 5 mmol·L^-^¹), and high Mg (25 mmol·L^-^¹). Each treatment consisted of 50 tobacco plants for each cultivar.

A hydroponic cultivation system with artificial light sources was employed. The light intensity was maintained at 4950 lx, air temperature at 25°C, photoperiod at 14 h d^-^¹, and relative humidity at 55–60%. Uniform tobacco seedlings at the seven-leaf and one-heart stage were selected and transplanted into hydroponic containers with a capacity of 2000 mL, with one plant per container. All seedlings were initially cultivated in full-strength Hoagland nutrient solution for 10 days to ensure acclimation. Subsequently, the nutrient solution was replaced with Hoagland solution containing the designated Mg concentrations for each treatment. The nutrient solution was renewed every 3 days, and the pH was adjusted and maintained at approximately 6.0. Aeration was provided using an air pump for 1 h per day to ensure sufficient dissolved oxygen in the solution.

### 2.2 Measurement items and methods

#### 2.2.1 Measurement of spectral reflectance

**Figure 1:**
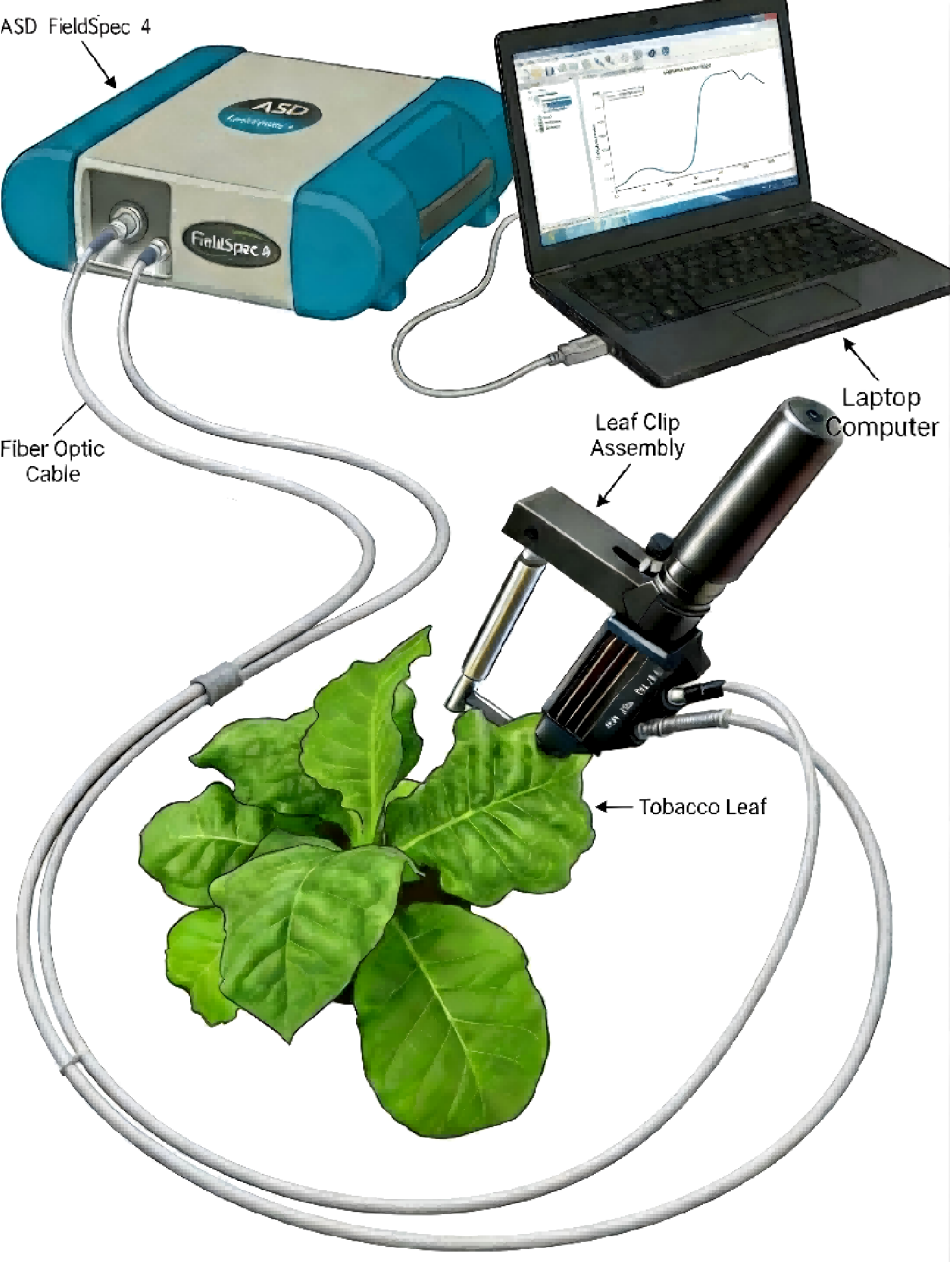
Spectral reflectance measurement.

On the 10th, 20th, 30th, 40th, and 50th days after the application of magnesium treatments, considering the rapid growth cycle and nutrient sensitivity of flue-cured tobacco under hydroponic conditions, an ASD FieldSpec 4 spectroradiometer (Analytical Spectral Devices, Boulder, CO, USA), with a spectral range of 350–2500 nm, was used to collect leaf spectral reflectance data from fresh tobacco leaves, as shown in **Figure 1**. For each treatment, nine representative plants were selected for spectral measurements. Using a standard leaf clip assembly, spectral reflectance was measured at five positions on each leaf, namely the leaf apex, near-apex region, middle leaf region, near-base region, and leaf base, by firmly clamping the leaf to ensure consistent optical geometry. At each position, areas with major veins, disease symptoms, or mechanical damage were deliberately avoided to minimize noise interference. Ten consecutive spectral scans were acquired using the fiber-optic probe, and the mean reflectance spectrum was calculated to reduce measurement noise. Consequently, 45 spectral samples (9 plants × 5 positions) were obtained for each sampling period, resulting in a total of 225 spectral samples across all growth stages.

#### 2.2.2 Measurement of magnesium content

After completion of leaf spectral measurements, the same leaves used for spectral acquisition were sampled from each treatment. The collected leaf samples were immediately blanched to deactivate enzymatic activity, followed by oven-drying to constant weight, fine grinding, and sieving through a 60-mesh screen. Subsequently, the powdered samples were subjected to wet digestion using a hydrogen peroxide–nitric acid (H_2_O_2_–HNO_3_) digestion protocol. The magnesium (Mg) concentration in the tobacco leaf samples was then determined using inductively coupled plasma optical emission spectrometry (ICP–OES) with an Agilent ICP–OES system (Agilent Technologies, Santa Clara, CA, USA) (Zhang et al., 2009).

### 2.3 Spectral preprocessing

#### 2.3.1 FD

Derivative spectroscopy, as a commonly used preprocessing technique in hyperspectral data analysis, can effectively reduce baseline effects and background interference caused by soil background, surface impurities, and atmospheric conditions. By enhancing subtle spectral features and separating overlapping absorption information, derivative transformation improves the discrimination of target spectral characteristics and enhances the robustness and accuracy of subsequent monitoring models, thereby facilitating the extraction of plant physiological information (Yu et al., 2013). In this study, the FD of the original hyperspectral reflectance was calculated using the following formula Equation 1:

(1) $$\begin{array}{c} R^{\prime }\left(\lambda_{i}\right)=\frac{R\left(\lambda_{i+1}\right)-R\left(\lambda_{i-1}\right)}{\lambda_{i+1}+\lambda_{i-1}} \end{array}$$

Where ${\text{λ}}_{\text{i}}$: Denotes the wavelength at band i;

$R\;(\lambda_{i})$: Represents the original spectral reflectance at wavelength ${\text{ λ}}_{\text{i}}$;

${\text{ R}}^{"}\left({\text{λ}}_{\text{i}}\right)$: Denotes the first derivative reflectance at wavelength ${\text{ λ}}_{\text{i}}$.

#### 2.3.2 SNV

SNV transformation is a scatter-correction method that standardizes individual spectra by subtracting the mean reflectance value and dividing by the standard deviation across all bands within a single spectrum. This approach is primarily used to minimize multiplicative scattering effects and additive baseline shifts caused by variations in illumination intensity, sensor–target distance, and surface roughness (Jinyuan and Haitao, 2017). SNV enhances spectral comparability, suppresses redundant information, and emphasizes subtle spectral differences while reducing noise interference.

The SNV transformation is defined as Equation 2:

(2) $$\begin{array}{c} SNV_{i,k}=\frac{X_{i,k}-{\bar{X}}_{i}}{s_{i}} \end{array}$$

Where $X_{i,k\;}$ is the original reflectance of the i-th sample at the k-th wavelength;

${\bar{X}}_{i}$ is the mean reflectance of the i-th sample across all wavelengths;

$s_{i}$ is the standard deviation of the reflectance values of the i-th sample;

i=1,2,…,n denotes the sample index;

k=1,2,…,m denotes the wavelength band index.

#### 2.3.3 MSC

MSC is a widely used spectral preprocessing technique designed to correct scattering effects caused by particle size variation, surface heterogeneity, and optical path length differences, thereby improving the signal-to-noise ratio (SNR) of hyperspectral data (Linfeng et al., 2024).

The MSC procedure was implemented as follows:

① The mean spectrum $\bar{X}$ of the calibration dataset was calculated and used as a reference spectrum. Each sample spectrum ${\text{X}}_{\text{i}}$ was then linearly regressed against the reference spectrum Equation 3:

(3) $$\begin{array}{c} X_{i}=a_{i}+b_{i}\bar{X} \end{array}$$

② The regression coefficients $a_{i}$(offset) and $b_{i}$(slope) were estimated using the least squares method. The original spectrum was subsequently corrected using Equation 4:

(4) $$\begin{array}{c} X_{i,MSC}=\frac{X_{i}-a_{i}}{b_{i}} \end{array}$$

③ Through this transformation, multiplicative and additive scattering effects were effectively removed, allowing the corrected spectra to better represent the intrinsic chemical absorption characteristics of the samples.

### 2.4 Feature variable selection

Hyperspectral data are characterized by high dimensionality, strong collinearity among adjacent spectral bands, and substantial information redundancy (Cheng and Hu, 2001). Appropriate feature variable selection methods are therefore essential to reduce spectral redundancy, alleviate model complexity, and improve model robustness and generalization performance. In this study, three representative feature variable selection algorithms—CARS, SPA, and GA—were employed for spectral variable optimization.

The CARS algorithm integrates Monte Carlo sampling with PLSR to iteratively select informative wavelengths. During each sampling process, wavelengths are adaptively retained or eliminated according to the absolute values of the PLSR regression coefficients, following an exponential decay strategy. Subsequently, cross-validation is applied to identify the optimal subset of wavelengths that contribute most significantly to the prediction of physicochemical parameters, thereby yielding a parsimonious and informative variable set (Linfeng et al., 2024).

The SPA is a forward variable selection technique designed to minimize multicollinearity among spectral variables. SPA selects candidate bands by projecting each spectral variable onto the orthogonal subspace of previously selected variables and evaluating the magnitude of the projection vectors. Variables with the largest projection contribution and minimal redundancy are sequentially selected, and the final feature subset is determined based on the performance of a calibration model, effectively reducing collinearity while preserving relevant spectral information (Li et al., 2020b).

The GA is a global optimization method inspired by biological evolution mechanisms, including natural selection, crossover, and mutation. GA operates directly on encoded spectral variable subsets without requiring assumptions of continuity or differentiability of the objective function. Owing to its implicit parallelism and strong global search capability, GA employs a probabilistic optimization strategy to iteratively evolve candidate solutions, enabling adaptive exploration of the search space and identification of near-optimal spectral variable combinations for model construction (Shu et al., 2024).

### 2.5 Modeling methods

To ensure sufficient model training while minimizing the risk of overfitting, models were developed separately for each sampling period. For each period, the 45 spectral samples were randomly divided into training and testing sets at a ratio of 7:3. Model hyperparameters were optimized exclusively within the training set using five-fold cross-validation (K = 5), ensuring that the testing data remained independent for performance evaluation.

#### 2.5.1 PLSR

PLSR is a multivariate statistical modeling technique widely used to explore the relationships between multiple correlated independent variables and one or more dependent variables. It constructs regression models by projecting the predictor variables and response variables onto a new latent variable space, in which the covariance between the independent and dependent variables is maximized (Haaland and Thomas, 1988). By retaining the latent variables that explain the maximum shared variance, PLSR effectively reduces data dimensionality, mitigates multicollinearity, suppresses noise, and extracts the most relevant information for explaining the dependent variable. Owing to these advantages, PLSR is particularly suitable for high-dimensional and strongly correlated hyperspectral data.

#### 2.5.2 ELM

ELM is a single-hidden-layer feedforward neural network characterized by its fast learning speed and strong nonlinear fitting capability. In ELM, the connection weights between the input layer and the hidden layer, as well as the hidden-layer biases, are randomly assigned and remain fixed during training, and only the output weights are estimated analytically. This mechanism significantly reduces computational complexity while maintaining satisfactory generalization performance. In this study, the Sigmoid function was adopted as the activation function of the hidden layer, owing to its stability and suitability for nonlinear regression tasks (Li et al., 2020b).

#### 2.5.3 SVR

Support Vector Regression (SVR) is a supervised learning algorithm derived from statistical learning theory, which employs kernel functions to map input variables from the original space into a high-dimensional feature space, enabling the modeling of complex nonlinear relationships. SVR determines an optimal regression function by minimizing a regularized objective function based on an ϵ-insensitive loss function, which balances prediction accuracy and model complexity. The selection of appropriate kernel functions and regularization parameters is critical for achieving optimal performance. Furthermore, SVR effectively suppresses the influence of outliers through margin constraints, thereby enhancing model robustness and stability. In hyperspectral applications, SVR has been widely used for both linear and nonlinear regression problems due to its strong generalization capability (Lin et al., 2020).

#### 2.5.4 RBF

The radial basis function (RBF) neural network is a typical three-layer feedforward artificial neural network consisting of an input layer, a hidden layer, and an output layer. Neurons in the hidden layer use radial basis functions as activation functions, enabling nonlinear mapping through localized responses of input samples in the feature space. The RBF network can adaptively approximate complex nonlinear relationships by adjusting the number of hidden layer nodes and the spread parameters of the radial basis functions. Due to its local approximation mechanism, the RBF network exhibits fast convergence, a relatively simple structure, and low sensitivity to initial parameters, resulting in strong nonlinear modeling capability. In hyperspectral data modeling, the RBF network effectively captures nonlinear relationships between spectral features and physicochemical indicators, making it suitable for quantitative inversion of continuous variables (Jia, 2010).

### 2.6 Model evaluation

The coefficient of determination (R²) is used to evaluate the goodness of fit of the monitoring model and quantify the degree to which the independent variables explain the variability of the dependent variable. Its value ranges from 0 to 1 (Wang et al., 2024). A higher R² value indicates that the predicted values are more closely and uniformly distributed around the fitted regression line, reflecting superior model performance. The calculation of R² is shown in Equation 5:

(5) $$R^{2}\;=\;\frac{\sum_{i=1}^{n}{\left(y_{mi}-y_{pi}\right)}^{2}}{\sum_{i=1}^{n}{\left(y_{mi}-y_{mean}\right)}^{2}}$$

where $y_{pi}$and $y_{mi}$represent the predicted and measured values of the *i*-th sample, respectively; $y_{mean}$denotes the mean of the measured values; and n is the total number of samples in the dataset (Liu et al., 1984).

The Root Mean Square Error (RMSE) is used to quantify the deviation between predicted and measured values and serves as an important indicator of prediction accuracy in machine learning models. A lower RMSE value indicates smaller prediction errors and higher model accuracy, whereas a higher RMSE suggests poorer predictive performance. The RMSE is calculated as shown in Equation 6:

(6) $$RMSE\sqrt{\frac{1}{n}\sum_{i=1}^{n}{\left(y_{pi}-y_{mi}\right)}^{2}}$$

where $y_{pi}$ and $y_{mi}$ denote the predicted and measured values of the *i*-th sample, respectively, and n represents the number of samples (Liu et al., 1984; Li et al., 2020a).

## 3 Results

### 3.1 Magnesium content in different varieties of flue-cured tobacco at different growth stages

**Figure 2:**
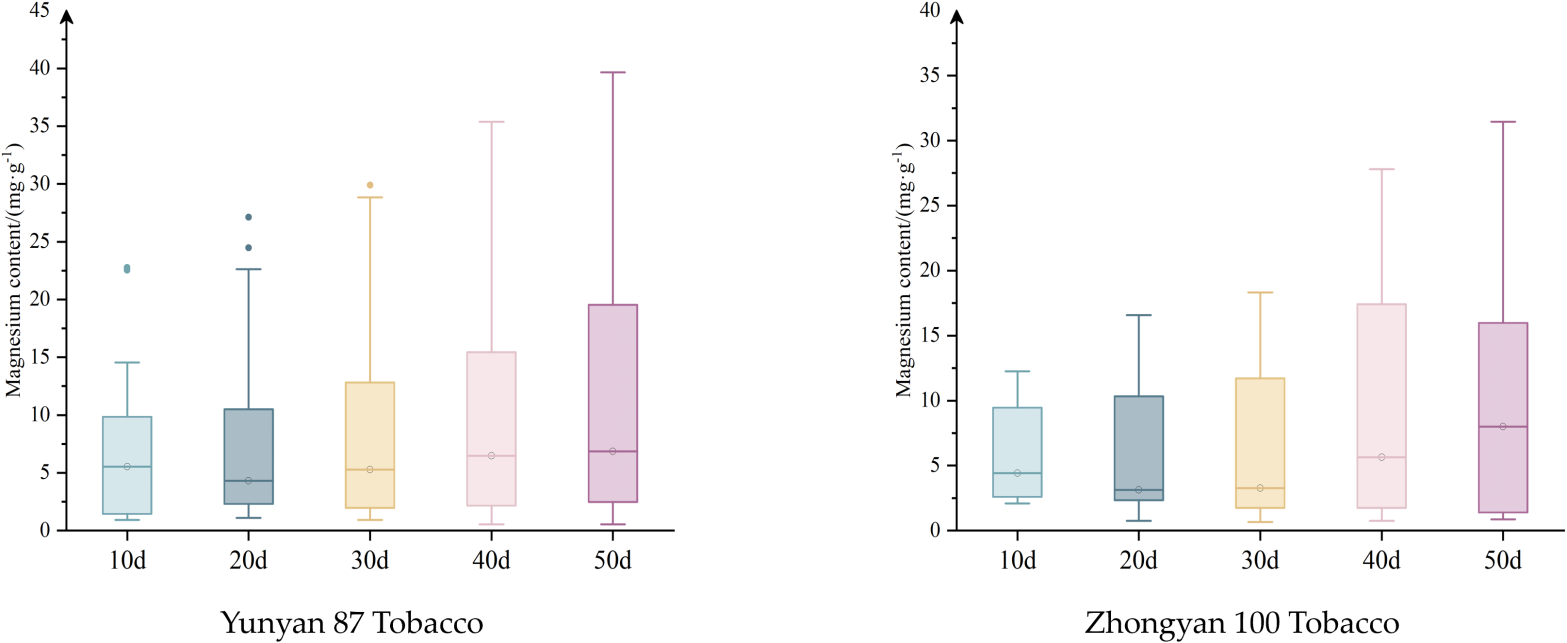
Distribution of Magnesium content at different periods.

**Figure 2** presents the distribution of magnesium content in tobacco leaves at dif-ferent periods. As shown in **Figure 2**, significant differences in leaf magnesium content are observed among different periods. With increasing time, the differences in magnesium content between Yunyan 87 and Zhongyan 100 gradually increase. At the same period, the variation in magnesium content of Yunyan 87 is greater than that of Zhongyan 100, particularly at 40 and 50 days after the application of different magnesium concentrations. At 40 days, the magnesium content of Yunyan 87 ranges from 0.54 to 35.37 mg·g^-^¹, whereas that of Zhongyan 100 ranges from 0.76 to 27.80 mg·g^-^¹. At 50 days, the magnesium content ranges are 0.55–39.66 mg·g^-^¹ for Yunyan 87 and 0.86–31.46 mg·g^-^¹ for Zhongyan 100, respectively.

### 3.2 Correlation between magnesium content in different periods and different spectral pretreatments

**Figure 3:**
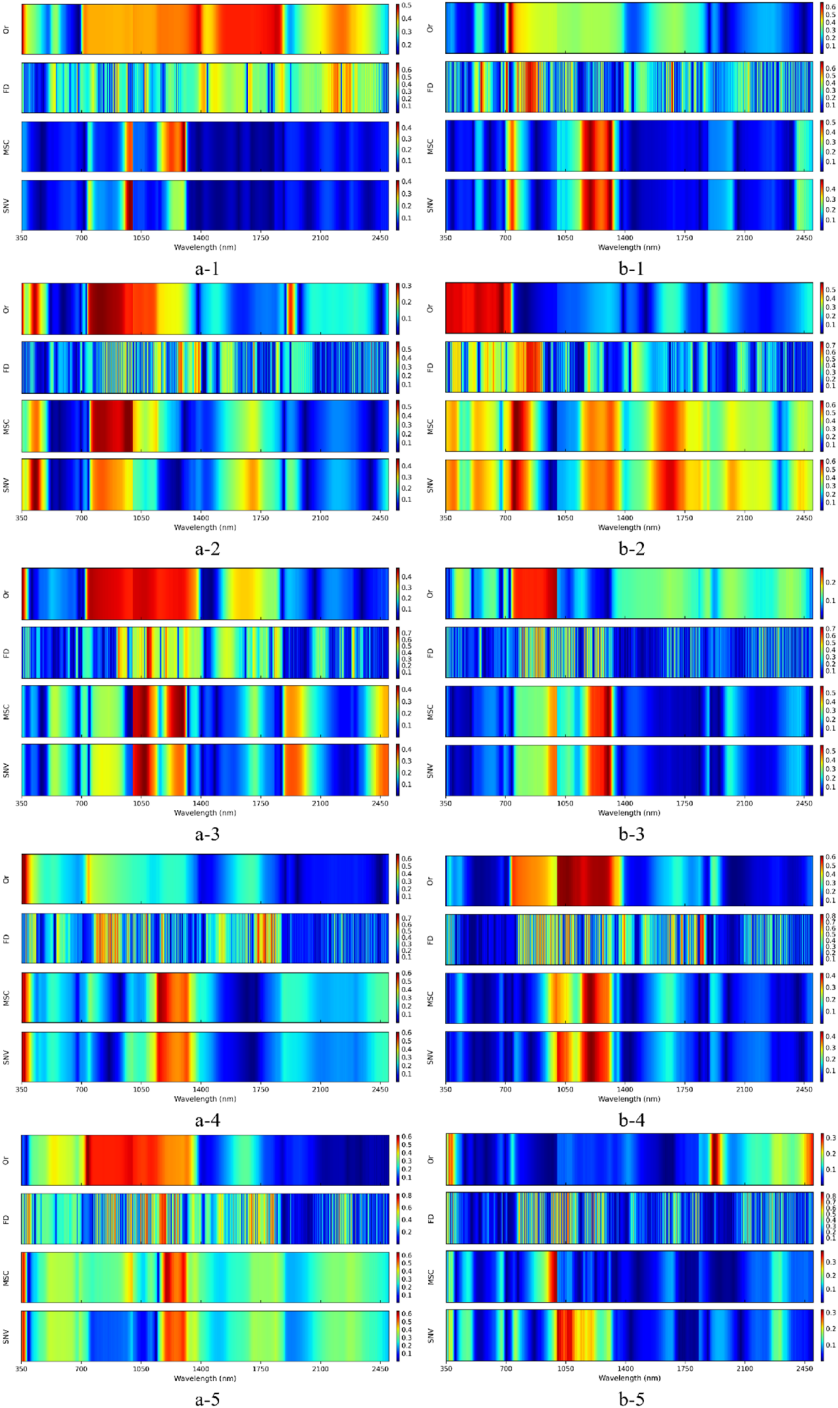
Correlation between spectral reflectance of tobacco leaves and magnesium content. a-1 to a-5 represent Yunyan 87 with different magnesium concentrations added for 10,20,30,40, and 50 days; b-1 to b-5 represent Zhongyan 100 with the same treatment periods.

To identify sensitive wavebands for estimating magnesium content in flue-cured tobacco at different periods, Pearson correlation analysis was performed between the pretreated spectral reflectance and the measured magnesium content. **Figure 3** shows the absolute values of the Pearson correlation coefficients between spectral reflectance at each wavelength and magnesium content. After applying first derivative (FD), multiplicative scatter correction (MSC), and standard normal variate (SNV) preprocessing to the original spectra (Or), the absolute correlation coefficients were markedly enhanced across all periods.

As shown in **Figure 3**, at 10 days, the maximum absolute correlation coefficients between magnesium content and pretreated spectra for both cultivars exceeded 0.40, with ranges of 0.41–0.71 for Yunyan 87 and 0.49–0.70 for Zhongyan 100. The FD-pretreated spectra exhibited a pronounced saw-tooth pattern, indicating alternating high and low correlations across adjacent wavebands. The strongest correlations were observed at 1001 nm for Yunyan 87 and 853 nm for Zhongyan 100, with absolute correlation coefficients of 0.71 and 0.70, respectively.

At 20 days, following spectral preprocessing, the absolute correlation coefficients ranged from 0.46 to 0.59 for Yunyan 87 and from 0.58 to 0.76 for Zhongyan 100. The maximum correlations after FD preprocessing occurred at 1271 nm and 830 nm, with absolute correlation coefficients of 0.59 and 0.76, respectively.

At 30 days, the strongest correlations between magnesium content and FD-pretreated spectra were observed within the wavelength ranges of 1050–1300 nm for Yunyan 87 and 850–1200 nm for Zhongyan 100, with maximum absolute correlation coefficients of 0.80 and 0.72, respectively.

At 40 days, the absolute correlation coefficients ranged from 0.60 to 0.74 for Yunyan 87 and from 0.42 to 0.82 for Zhongyan 100. After FD preprocessing, magnesium content showed strong correlations at 1779 nm and 918 nm, with absolute correlation coefficients of 0.74 and 0.82, respectively.

At 50 days, the absolute correlation coefficients between magnesium content and the FD-, MSC-, and SNV-pretreated spectra of Yunyan 87 were 0.84, 0.64, and 0.63, respectively, with the strongest correlation occurring at 899 nm after FD preprocessing. For Zhongyan 100, the strongest correlation was observed at 1067 nm following FD preprocessing, with an absolute correlation coefficient of 0.87.

Overall, spectral preprocessing substantially enhanced the correlation between hyperspectral reflectance and magnesium content. The correlation strength increased with time, reaching maximum values at 50 days, particularly after FD preprocessing. In addition, the correlations obtained for Yunyan 87 were consistently higher than those for Zhongyan 100.

### 3.3 Selection of the optimal pretreatment method

Partial Least Squares Regression (PLSR) was employed to evaluate the effects of different spectral pretreatment methods on model performance. **Table 1** summarizes the PLSR models developed for estimating magnesium content in Yunyan 87 and Zhongyan 100 tobacco leaves at different periods using spectral data subjected to various pretreatment methods.

**Table 1:** Model fitting results obtained using different spectral pretreatment methods.

Treatment time	Variety	Preprocessing method	Training set		Test set	
			R^2^	RMSE (mg·g^-1^)	R^2^	RMSE (mg·g^-1^)
10d	Yunyan 87	FD	0.80	3.73	0.27	5.44
		MSC	0.74	2.96	0.43	4.03
		SNV	0.90	2.04	0.57	3.91
	Zhongyan 100	FD	0.93	0.93	0.42	3.50
		MSC	0.55	2.38	0.25	3.84
		SNV	0.56	2.59	0.26	3.50
20d	Yunyan 87	FD	0.82	3.94	0.69	9.05
		MSC	0.73	4.61	0.46	11.25
		SNV	0.68	5.36	0.44	10.49
	Zhongyan 100	FD	0.90	1.44	0.60	2.90
		MSC	0.78	2.32	0.36	5.44
		SNV	0.66	2.90	0.24	5.26
30d	Yunyan 87	FD	0.94	2.52	0.58	10.61
		MSC	0.76	4.44	0.68	9.39
		SNV	0.69	6.34	0.42	11.00
	Zhongyan 100	FD	0.90	3.16	0.50	3.87
		MSC	0.62	5.07	0.56	3.20
		SNV	0.74	4.95	0.60	2.39
40d	Yunyan 87	FD	0.89	4.09	0.52	8.99
		MSC	0.72	6.00	0.62	7.34
		SNV	0.71	6.28	0.61	7.68
	Zhongyan 100	FD	0.96	1.84	0.45	7.03
		MSC	0.98	1.33	0.59	6.48
		SNV	0.93	2.35	0.79	4.80
50d	Yunyan 87	FD	0.92	3.72	0.48	9.87
		MSC	0.83	5.71	0.58	8.2
		SNV	0.74	6.85	0.79	6.07
	Zhongyan 100	FD	0.72	5.03	0.45	6.71
		MSC	0.92	2.55	0.67	5.88
		SNV	0.69	5.26	0.36	7.32

As shown in **Table 1**, for Yunyan 87 at 10 and 50 days after the application of different magnesium concentrations, and for Zhongyan 100 at 30 and 40 days, standard normal variate (SNV) preprocessing yielded the best model performance, with test set R² values exceeding 0.57. For Yunyan 87 at 20 days, and for Zhongyan 100 at 10 and 20 days, the models based on first-derivative (FD) preprocessed spectra exhibited the highest predictive accuracy, with test set R² values greater than 0.42. For Yunyan 87 at 30 and 40 days, and for Zhongyan 100 at 50 days, multiplicative scatter correction (MSC) was identified as the optimal pretreatment method, achieving test set R² values above 0.62.

Subsequent analyses were conducted using the spectra preprocessed with the optimal methods identified above.

### 3.4 Feature waveband extraction

**Figure 4:**
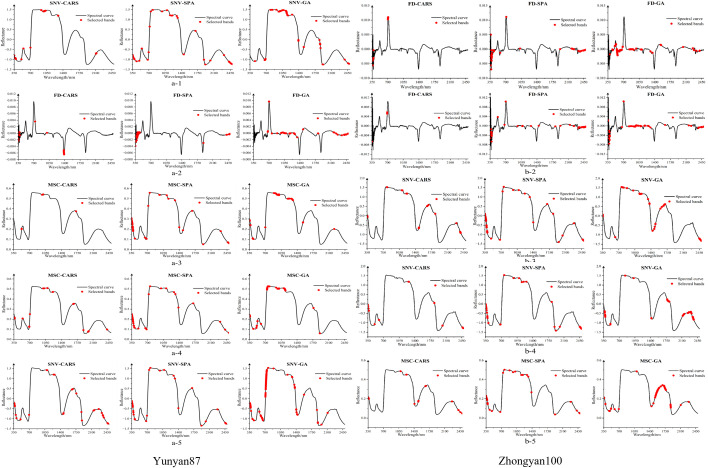
Selection results of characteristic spectral bands of samples at different periods.

To reduce modeling complexity and eliminate redundant information, Competitive Adaptive Reweighted Sampling (CARS), the Successive Projections Algorithm (SPA), and the Genetic Algorithm (GA) were employed to select sensitive wavebands. **Figure 4** illustrates the distribution of characteristic wavebands selected from the spectral samples of Yunyan 87 and Zhongyan 100 at different periods, with distinct wavelength positions identified by each algorithm.

For Yunyan 87, at 10, 20, 30, and 40 days after the application of different magnesium concentrations, the near-infrared (NIR) region accounted for the largest proportion of the selected feature wavebands, representing 86%, 90%, 93%, and 65% of the total bands, respectively. In contrast, at 50 days, the proportion of feature wavebands in the visible region increased markedly to 45%, whereas the NIR proportion decreased to 37%. Across all periods, the CARS algorithm predominantly selected wavebands in the NIR region. The SPA algorithm extracted more wavebands from the ultraviolet region at 20, 30, 40, and 50 days, while at 10 days it mainly selected NIR wavebands. The GA algorithm primarily selected NIR wavebands at 10–40 days, whereas visible-region wavebands dominated at 50 days.

For Zhongyan 100, the distribution of feature wavebands differed markedly from that of Yunyan 87. At 10 days, the visible region contributed the highest proportion of selected wavebands, accounting for 62% of the total. At 20, 30, 40, and 50 days, feature wavebands were predominantly selected from the NIR region, representing 68%, 59%, 82%, and 65%, respectively. For the CARS algorithm, visible-region wavebands accounted for 76.9% and 100% at 10 and 20 days, respectively, whereas NIR wavebands dominated from 30 to 50 days. The SPA algorithm selected visible-region wavebands at proportions of 40%, 77.5%, 72.5%, and 80% at 10, 30, 40, and 50 days, respectively. The GA algorithm consistently selected a high proportion of NIR wavebands across all periods, reaching a maximum of 82% at 40 days.

Overall, for Yunyan 87, feature wavebands were mainly distributed in the NIR region from 10 to 40 days, while the contribution of the visible region increased at 50 days. In contrast, for Zhongyan 100, feature wavebands were primarily concentrated in the visible region at 10 days and remained stably distributed in the NIR region from 20 to 50 days.

### 3.5 Analysis of magnesium content monitoring models based on feature extraction

**Figure 5:**
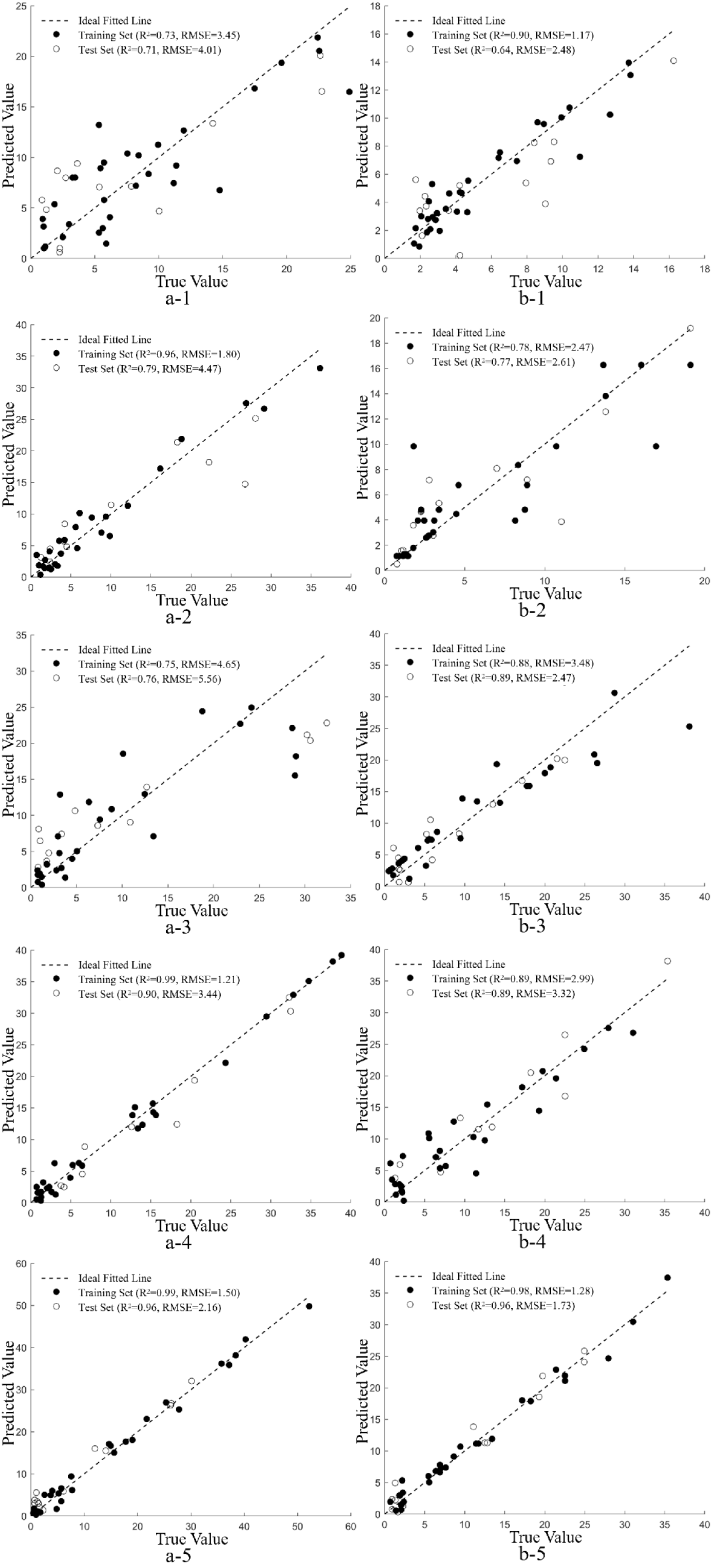
Scatter plot of predicted versus observed magnesium contents based on the model.

Based on the sensitive wavebands screened in **Figure 4**, regression models were constructed using SVR, ELM, and RBF neural network algorithms. The modeling results are presented in **Tables 2**, **3**, **Figure 5**. As shown in **Tables 2**, **3**, at 10, 20, 30, 40, and 50 days after the application of different magnesium concentrations, the optimal feature extraction algorithms for Yunyan 87 were CARS, CARS, GA, CARS, and CARS, respectively. In contrast, regression models based on SPA feature extraction consistently showed the poorest performance across all periods, indicating that the SPA feature extraction method is not suitable for constructing hyperspectral monitoring models of magnesium content in Yunyan 87.

**Table 2:** Model fitting results of magnesium content for Yunyan 87 tobacco leaves.

Treatment time	Variety	Preprocessing method	Modeling method	Training set		Test set	
				R^2^	RMSE (mg·g^-1^)	R^2^	RMSE (mg·g^-1^)
10d	SNV	CARS	SVR	0.87	2.70	0.61	5.30
			ELM	0.73	3.45	0.71	4.01
			RBF	0.93	1.20	0.68	5.35
		SPA	SVR	0.91	1.92	0.36	6.3
			ELM	0.79	3.14	0.2	6.49
			RBF	0.84	2.93	0.48	4.28
		GA	SVR	0.92	2.09	0.63	3.75
			ELM	0.86	2.69	0.65	6.00
			RBF	0.87	2.51	0.57	4.13
20d	FD	CARS	SVR	0.91	3.00	0.70	5.78
			ELM	0.83	3.75	0.50	6.29
			RBF	0.96	1.80	0.79	4.47
		SPA	SVR	0.87	2.99	0.19	11.43
			ELM	0.79	3.14	0.64	5.28
			RBF	0.70	4.04	0.52	6.92
		GA	SVR	0.70	2.66	0.53	6.75
			ELM	0.89	2.28	0.47	6.92
			RBF	0.99	0.89	0.59	6.84
30d	MSC	CARS	SVR	0.80	4.43	0.53	7.08
			ELM	0.72	5.58	0.63	3.99
			RBF	0.86	4.09	0.36	5.75
		SPA	SVR	0.59	6.86	0.27	6.95
			ELM	0.46	6.95	0.18	11.31
			RBF	0.97	1.63	0.33	8.17
		GA	SVR	0.74	5.45	0.62	5.06
			ELM	0.75	4.61	0.76	5.65
			RBF	0.84	3.84	0.64	6.39
40d	MSC	CARS	SVR	0.91	3.65	0.79	4.87
			ELM	0.76	4.37	0.52	6.88
			RBF	0.99	1.21	0.90	3.44
		SPA	SVR	0.79	4.83	0.55	9.30
			ELM	0.64	7.29	0.64	6.11
			RBF	0.95	2.66	0.55	7.37
		GA	SVR	0.68	6.78	0.52	7.61
			ELM	0.50	8.14	0.42	9.19
			RBF	0.60	7.63	0.33	8.67
50d	SNV	CARS	SVR	0.90	4.19	0.86	5.47
			ELM	0.86	4.12	0.70	5.89
			RBF	0.99	1.50	0.96	2.16
		SPA	SVR	0.97	2.53	0.39	12.38
			ELM	0.99	1.64	0.30	13.06
			RBF	0.98	2.02	0.62	7.33
		GA	SVR	0.87	5.07	0.41	8.68
			ELM	0.97	3.66	0.72	9.46
			RBF	0.87	4.70	0.82	5.95

**Table 3:** Model fitting results of magnesium content for Zhongyan 100 Tobacco leaves.

Treatment time	Variety	Preprocessing method	Modeling method	Training set		Test set	
				R^2^	RMSE(mg·g^-1^)	R^2^	RMSE(mg·g^-1^)
10d	FD	CARS	SVR	0.81	1.74	0.48	2.47
			ELM	0.68	2.32	0.47	1.84
			RBF	0.52	2.83	0.46	2.37
		SPA	SVR	0.90	1.28	0.30	2.88
			ELM	0.41	3.08	0.26	3.38
			RBF	0.90	1.17	0.64	2.48
		GA	SVR	0.90	0.17	0.41	2.23
			ELM	0.46	1.93	0.19	4.01
			RBF	0.32	3.50	0.48	1.99
20d	FD	CARS	SVR	0.41	3.96	0.13	3.51
			ELM	0.78	2.47	0.77	2.61
			RBF	0.33	4.39	0.21	3.27
		SPA	SVR	0.99	0.29	0.45	3.81
			ELM	0.38	4.06	0.28	4.80
			RBF	0.92	1.42	0.63	2.88
		GA	SVR	0.89	1.60	0.64	3.00
			ELM	0.75	2.53	0.70	3.25
			RBF	0.45	3.98	0.38	4.44
30d	SNV	CARS	SVR	0.98	1.18	0.67	6.13
			ELM	0.85	1.82	0.70	2.78
			RBF	0.90	2.84	0.84	3.69
		SPA	SVR	0.72	4.61	0.57	6.30
			ELM	0.88	1.32	0.54	3.63
			RBF	0.59	6.16	0.68	4.59
		GA	SVR	0.88	3.48	0.89	2.47
			ELM	0.99	1.04	0.29	8.70
			RBF	0.99	0.71	0.61	6.16
40d	SNV	CARS	SVR	0.94	2.37	0.80	3.72
			ELM	0.89	2.99	0.89	3.32
			RBF	0.95	2.36	0.76	3.59
		SPA	SVR	0.79	3.41	0.77	4.85
			ELM	0.25	8.15	0.19	9.2
			RBF	0.98	1.09	0.78	4.09
		GA	SVR	0.99	0.95	0.87	3.12
			ELM	0.69	4.61	0.83	3.26
			RBF	0.98	1.05	0.81	5.15
50d	MSC	CARS	SVR	0.95	2.23	0.75	3.74
			ELM	0.87	3.45	0.77	4.95
			RBF	0.98	1.28	0.96	1.73
		SPA	SVR	0.91	2.89	0.69	4.70
			ELM	0.98	1.05	0.53	5.15
			RBF	0.96	1.60	0.66	3.26
		GA	SVR	0.94	1.81	0.87	2.70
			ELM	0.95	1.11	0.62	3.43
			RBF	0.91	1.72	0.66	5.65

a-1, a-2, a-3, a-4, and a-5 represent Yunyan 87 treated with different magnesium concentrations for 10, 20, 30, 40, and 50 days, respectively; b-1, b-2, b-3, b-4, and b-5 represent Zhongyan 100 treated with different magnesium concentrations for 10, 20, 30, 40, and 50 days, respectively. The same notation applies hereinafter.

For Zhongyan 100, the optimal regression models at the five periods were established based on feature wavebands extracted by SPA, CARS, GA, CARS, and CARS, respectively, suggesting that the CARS feature extraction method is also applicable for constructing hyperspectral monitoring models of magnesium content in Zhongyan 100.

Using the optimal feature extraction, well-performing magnesium content monitoring models for Yunyan 87 were obtained using SVR, ELM, and RBF neural networks, with R² > 0.50 and RMSE< 6.39 mg·g^-^¹. For Zhongyan 100, the optimal modeling algorithms at the five periods were RBF, ELM, SVR, ELM, and RBF neural network, respectively.

**Figure 5** shows the scatter plots of predicted versus observed magnesium contents in flue-cured tobacco leaves at different periods based on the optimal monitoring models. The predicted and observed values are closely distributed around the 1:1 line, with few deviations, indicating good model fitting performance. As the duration of magnesium treatment increases, the overall performance of the magnesium monitoring models for both Yunyan 87 and Zhongyan 100 exhibited an improvement, with both models achieving their best performance at 50 days of magnesium treatment. For Yunyan 87, the test set R² was 0.96 and the RMSE was 2.16 mg·g^-^¹; for Zhongyan 100, the test set R² was 0.96 and the RMSE was 1.73 mg·g^-^¹. These results indicate stronger correlations between the spectral characteristics of tobacco leaves and magnesium content, as well as higher model prediction accuracy under prolonged magnesium treatment.

## 4 Discussion

Magnesium is a key nutrient element involved in chloroplast structure and the photosynthetic process. Variations in Mg content can effectively reflect the growth status of flue-cured tobacco and are closely associated with leaf yield and quality. At present, non-destructive monitoring of nutrient elements in flue-cured tobacco based on remote sensing technology has become one of the research hotspots (Diao et al., 2023). However, during the acquisition of spectral information, various types of noise and non-target information are often introduced due to sample heterogeneity, differences in instrument response, and interference from external environmental conditions. These factors can weaken the correlation between spectral data and physicochemical indicators and increase the difficulty of model construction. Therefore, the application of appropriate spectral preprocessing methods can significantly improve spectral data quality by removing noise, correcting baseline drift, and enhancing characteristic information, thereby providing a reliable foundation for subsequent modeling (Jinyuan and Haitao, 2017).

The result of this study indicate that the optimal spectral preprocessing methods corresponding to different growth stages vary among different flue-cured tobacco cultivars. This variation may be related to the physiological characteristics at different developmental stages and differences in cultivar sensitivity to magnesium. During growth stages with relatively small differences in Mg content, spectral signals are more susceptible to background interference, making it necessary to adopt preprocessing methods that can highlight subtle changes and improve spectral resolution. In contrast, during stages when spectra are strongly affected by internal leaf structure and light scattering, preprocessing methods that eliminate structural differences are required to enhance data comparability. In addition, different cultivars exhibit varying degrees of improvement in correlation under the same preprocessing conditions, indicating that cultivar factors influence the spectral response to changes in Mg content to a certain extent.

In terms of regression modeling, PLSR as a classical multivariate statistical method, has been widely applied in fields such as chemistry, economics, and bioinformatics due to its suitability for handling high-dimensional data and multicollinearity (Chen et al., 2017). In this study, PLSR was used to estimate Mg content in two flue-cured tobacco varieties; however, the prediction accuracy was relatively low, which may be attributed to severe redundancy among hyperspectral variables and the relatively limited sample size. To reduce model complexity, mitigate the influence of redundant information, and select subset of variables that have significant contributions to model performance, thereby improving predictive accuracy, reducing computational burden, and avoiding overfitting—three feature selection methods, namely CARS, SPA and GA, were further introduced to screen sensitive spectral bands (Jiang et al., 2025). The results showed that the characteristic bands selected by these methods were mainly concentrated in the near-infrared region, which is closely associated with crop nutrient status and internal leaf structure (Guo, 2019), and this finding was consistent with previous studies on hyperspectral estimation of magnesium content in rubber tree leaves, indicating the physiological and practical relevance of the selected bands (Chen et al., 2022).

In machine learning modeling, nonlinear models such as ELM, SVR, and RBF neural network exhibited overall superior predictive performance compared with the traditional PLSR model when using the selected feature bands.

In machine learning modeling, nonlinear models such as ELM, SVR, and RBF neural network exhibited overall superior predictive performance compared with the traditional PLSR model when using the selected feature bands (Linfeng et al., 2024). This result indicated that machine learning approaches had clear advantages in capturing the complex nonlinear relationships between hyperspectral data and magnesium content. Moreover, the optimal combinations of feature bands and modeling methods varied across different growth stages, suggesting that the spectral response mechanisms of magnesium content differed throughout tobacco development. As growth progressed, the prediction accuracy of all models generally increased, and during later growth stages, the coefficient of determination (R²) for the test sets of some models exceeded 0.90. This phenomenon may be related to the gradual accumulation of physicochemical differences in tobacco leaves under different magnesium treatments over time, which enhanced the detectability of magnesium-related spectral signals.

With respect to varietal differences, the predictive accuracy of the models varied between tobacco varieties, with Yunyan 87 generally outperforming Zhongyan 100. This discrepancy may be associated with differences in leaf structure, optical properties, and sensitivity to magnesium nutrition among varieties. Previous studies have demonstrated that variations in leaf thickness, chlorophyll content, and tissue structure can significantly affect spectral reflectance characteristics, thereby influencing the performance of nutrient inversion models (Guo, 2019). Compared with previous studies that have predominantly focused on nitrogen and potassium monitoring in flue-cured tobacco, this study provided new technical insights into magnesium content monitoring. From the perspective of spectral response mechanisms, magnesium deficiency is characterized by typical interveinal chlorosis, which differs from the symptoms of nitrogen and potassium deficiency and significantly alters multiple scattering processes within the leaf. The near-infrared feature bands identified in this study effectively captured spectral response differences induced by mesophyll structural degradation. Furthermore, unlike traditional single-stage monitoring approaches, this study revealed the nonlinear temporal characteristics of magnesium inversion accuracy over time and compared the stability of magnesium monitoring models between Yunyan 87 and Zhongyan 100.

## 5 Conclusions

This study systematically evaluated the correlation between magnesium content and hyperspectral information in two flue-cured tobacco varieties, Yunyan 87 and Zhongyan 100, at different periods. By employing three preprocessing methods and feature waveband selection techniques, various modeling approaches were constructed to estimate magnesium content, leading to the following main conclusions:

1. The optimal preprocessing methods differed between the two varieties across periods. As the periods progressed, the correlation between hyperspectral data and magnesium content significantly increased. Notably, at 50 days after treatment with different magnesium concentrations, the correlation coefficients between hyperspectral data preprocessed using the First Derivative (FD) method and the magnesium content of Yunyan 87 and Zhongyan 100 reached 0.84 and 0.87, respectively.
2. Based on the optimal preprocessing, the performance of feature waveband selection and modeling methods varied among periods and varieties. Overall, model fitting accuracy improved over time, particularly at 40 and 50 days, when the coefficients of determination (R²) for all prediction models exceeded 0.90. The prediction accuracy of models for Yunyan 87 was consistently higher than that for Zhongyan 100.

This study establishes a theoretical and technical foundation for monitoring magnesium content in flue-cured tobacco at different growth stages using spectral remote sensing, providing scientific support for precision fertilization and dynamic nutrient management in the field. In future research, multi-site experiments will be conducted across major flue-cured tobacco–producing regions in China, covering different soil types (such as calcareous soils, acidic soils, and sandy soils) and diverse climatic conditions. In addition, the effects of different cultivation and management practices will be integrated to systematically evaluate the stability and generalization ability of the proposed models under complex field conditions. This will further verify their practical applicability and provide a basis for the broader application of hyperspectral technology in the precision management of magnesium nutrition in flue-cured tobacco.

## Data Availability

The raw data supporting the conclusions of this article will be made available by the authors, without undue reservation.
