# Correction: Hyperspectral estimation of magnesium content in Yunyan 87 and Zhongyan 100 tobacco leaves using machine learning

**DOI:** 10.3389/fpls.2026.1842871

**Published:** 2026-04-14

**Authors:** Beibei Li, Qian Zhang, Zekai Wang, Yike Li, Hanjun Zhou, Xiefeng Ye

**Affiliations:** 1 Key Laboratory of Tobacco Cultivation of Tobacco Industry, National Tobacco Cultivation and Physiology and Biochemistry Research Centre, Tobacco Science College of Henan Agricultural University, Zhengzhou, China; 2 Bozhou Branch Company of Zunyi Tobacco Company, Zunyi, China

**Keywords:** flue-cured tobacco, hyperspectral remote sensing, machine learning models, magnesium content, non-destructive estimation

In the published article, there was an error in the title of Section 3.1. The title of Section 3.1 was incorrectly written as: “*Correlation between magnesium content in different periods and different spectral pretreatments*”.

A correction has been made to the section 3.1: “*Magnesium content in different varieties of flue-cured tobacco at different growth stages*”

The original version of this article has been updated.

